# Intraoperative Severe Anaphylactic Shock to Sugammadex in an Anesthesia-Naïve Patient

**DOI:** 10.7759/cureus.102320

**Published:** 2026-01-26

**Authors:** Alisa Wilkinson, Ziyad Knio, Alexander Metzger, Nabil Elkassabany, Jenna Leclerc

**Affiliations:** 1 Anesthesiology, University of Virginia, Charlottesville, USA; 2 Anesthesiology, Neuroscience, Division of Critical Care Medicine, University of Virginia, Charlottesville, USA

**Keywords:** allergy, anaphylaxis, case report, shock, sugammadex

## Abstract

Sugammadex is a revolutionary drug for the reversal of neuromuscular blocking agents (NMBAs). It is a modified gamma cyclodextrin that directly binds and inactivates steroidal nondepolarizing neuromuscular blockers to facilitate reversal with fewer adverse effects than previously used reversal agents. In general, literature supports its favorable safety profile, with common side effects being nausea, vomiting, headache, dry mouth, and dizziness. Sugammadex may also cause hypotension, bradycardia, or, rarely, anaphylaxis. The present case describes the clinical presentation and management of severe anaphylactic shock following intraoperative sugammadex administration to an anesthesia-naïve patient.

## Introduction

Neuromuscular blocking agents (NMBAs) have been used in anesthesiology practice for much of the last century [1]. These drugs are widely employed in general anesthetics to optimize intubating conditions and improve surgical exposure. NMBAs are generally categorized as either depolarizing or nondepolarizing according to their mechanism of action, with the use of nondepolarizing agents more common in current clinical practice [1]. 

Reversal of nondepolarizing NMBAs is necessary prior to emergence and extubation to promote the recovery of respiratory and laryngeal motor function. Prior to FDA approval of sugammadex in 2015, cholinesterase inhibitors (e.g., neostigmine) were the only reversal agents available for non-depolarizing NMBAs. Cholinesterase inhibitors function in an indirect manner to increase the concentration of acetylcholine at the neuromuscular junction to competitively antagonize the effect of non-depolarizing NMBAs on postsynaptic receptors. However, sugammadex is a new reversal agent with a novel mechanism; this modified gamma cyclodextrin directly encapsulates steroidal non-depolarizing NMBAs, immediately terminating their effect [2].

Compared to cholinesterase inhibitors, sugammadex has several important favorable aspects to support its current usage, including but not limited to faster and more predictable reversal of moderate neuromuscular blockade (indicated by lower variability in recovery times) [3], faster reversal of deep paralysis (including after rapid-sequence induction doses) [4], and significantly reduced incidence of residual neuromuscular blockade [5]. Additionally, in some scenarios, sugammadex can be a critical component to navigating out of a “cannot intubate, cannot ventilate” situation. Finally, some studies show that reversal with sugammadex is linked to a lower incidence of postoperative pulmonary complications [6], although the data are currently conflicting, with others showing no difference [7,8].

Sugammadex is generally considered to be safe with a limited side effect profile. It has been shown to have a lower risk of bradycardia (risk ratio (RR) 0.16) and postoperative nausea/vomiting (RR 0.52) compared to neostigmine [9]. In the 2015 FDA approval dataset, sugammadex was associated with hypotension (5%), vomiting (12%), bradycardia (1%), headache (5%), and nausea (26%) [10, 11]. Anaphylaxis, a rapid onset and severe multi-system hypersensitivity reaction, is the rarest but most life-threatening reaction to sugammadex, with an incidence estimated at 0.01% and 0.039%, and, importantly, can occur in patients without prior exposures [12].

We report here a case of severe intraoperative anaphylactic shock after routine administration of sugammadex to reverse a non-depolarizing NMBA in an anesthesia-naïve patient following elective inguinal hernia repair. A signed Health Insurance Portability and Accountability Act authorization to use and disclose existing information was obtained from the patient. This case report adheres to CARE Guidelines for Case Reports and did not require institutional review board approval.

## Case presentation

A 38-year-old male patient weighing 74 kg (BMI 29 kg/m²) with a medical history of type 2 diabetes mellitus (hemoglobin A1c 7.9%) presented for elective inguinal hernia repair. His preoperative anesthetic evaluation was otherwise unremarkable. The patient specifically denied prior anesthetic exposure and had no family history of anesthetic problems. 

Intraoperatively, the patient underwent an uneventful and hemodynamically stable induction with lidocaine, propofol, and 50 mg of rocuronium (at approximately 14:41). He was maintained on sevoflurane (average expired concentration of 2.0% throughout the case). Throughout the two-hour case, rocuronium was redosed twice (at 30 mg at 15:36 and 20 mg at 16:13). Mean arterial pressure (MAP) averaged 75 mmHg, with no pressor requirement. Pulse oximetry (SpO_2_) ranged from 98% to 100% on 50% inspired oxygen. Heart rate ranged from 60 to 120 beats per minute (bpm) with no electrocardiogram (EKG) abnormalities. 

**Figure 1:**
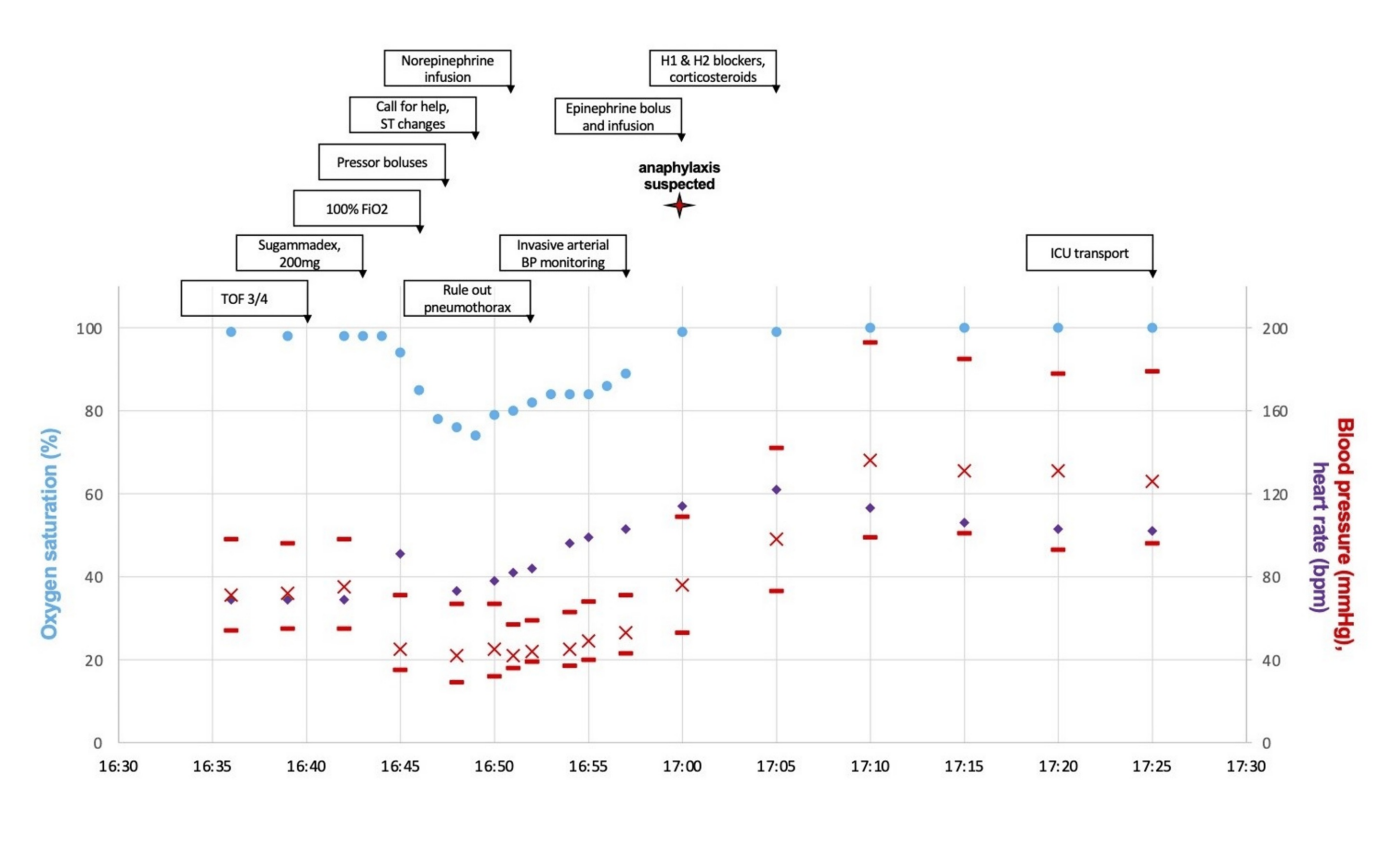
Time course of intraoperative events surrounding sugammadex anaphylactic period X axis: intraoperative time (in minutes); Y axis: scale ranging from 0 to 100; Red X: mean arterial pressure (mmHg); Upper red line: systolic blood pressure (mmHg); Lower red line: diastolic blood pressure (mmHg); Purple dot: heart rate (beats per minute (bpm)); Blue dot: oxygen saturation (% out of 100) TOF: train-of-four

Train-of-four monitoring revealed 3/4 twitches at 16:40, and the patient was administered 200 mg of sugammadex at 16:43. Within 3 minutes of sugammadex administration, the patient developed sustained severe hypoxia with SpO_2_ in the 70s (nadir 74%), increased airway pressures (37 from 14), and hypotension with MAPs in the 40s (nadir 42 mmHg) refractory to increased fresh gas flows, 100% fraction of inspired oxygen (FiO_2_), alveolar recruitment, pressure-driven intravenous crystalloid boluses (total 2 L), and high-dose vasopressors (2000 mcg phenylephrine, 150 mg ephedrine, and norepinephrine at 20 μg/min), and ST elevations were noted. Additional large-bore peripheral intravenous access and invasive arterial pressure monitoring were rapidly established. A focused ultrasound examination was performed to assess for lung and cardiac function, which was within normal limits aside from an underfilled heart. While initially cutaneous signs were absent, urticaria and angioedema were subsequently seen, and given the lack of recent administration of other agents, including NMBAs, antibiotics, or latex, the diagnosis was narrowed to suspected sugammadex-induced anaphylaxis. Immediate improvement in hemodynamics, other vital signs, and airway pressures was noted after a 100 μg epinephrine bolus and initiation of a 2 μg/min infusion. The patient concurrently received hydrocortisone, famotidine, and diphenhydramine [13]. Figure 1 provides the time course of intraoperative events during the anaphylactic period.

After the patient was stabilized intraoperatively, he was transferred to the surgical intensive care unit (SICU), intubated, and sedated with propofol and continued low-dose epinephrine infusion at 1 μg/min due to severe angioedema preventing extubation. Postoperative care in the SICU included scheduled intravenous dexamethasone, crystalloid fluid resuscitation (3L), and epinephrine infusion (initially continued at 1 μg/min and turned off on postoperative day (POD) #1). The patient was extubated at approximately 17 hours post sugammadex administration and discharged on POD #2. 

Supporting the diagnosis of severe anaphylactic shock, laboratory values were notable for a peak lactate of 4.01 mmol/L (normal 0.5-2.2 mmol/L) and tryptase of 83.8 ng/mL (normal 1-15 ng/mL) at 23 min and 75 min, respectively, after sugammadex administration. Lactate levels resolved (1.29) prior to discharge. Tryptase down-trended to 4.0 at 216 min and <1 ng/ml at approximately 15 hours post sugammadex administration.

## Discussion

This report describes the clinical presentation and management of confirmed severe anaphylactic shock following administration of sugammadex in an American Society of Anesthesiologists grade 2 anesthesia-naïve patient undergoing an elective inguinal hernia repair at a large academic institution. We describe the initial presentation of intraoperative undifferentiated shock, with subsequent narrowing of the differential to anaphylactic shock. 

Perioperative anaphylaxis is uncommon but often results in significant patient morbidity and mortality. The overall rate of perioperative anaphylaxis is reported at approximately one in 10,000 [14], with a rate of fatal or near-fatal anaphylaxis events of 1.26 in 100,000 procedures [15]. In 90% of cases, NMBAs, antibiotics, latex, chlorhexidine, and blue dye are identified as the inciting agents [12]. Accordingly, most cases of intraoperative anaphylaxis occur towards the beginning of an anesthetic. Anaphylaxis to sugammadex is quite rare (0.01% to 0.039%) in clinical practice and occurs at the end of an anesthetic when hemodynamics and vital signs are naturally rapidly fluctuating, a combination that often results in a significant delay in diagnosis [12]. Furthermore, no risk factors for sugammadex anaphylaxis have been identified, making it very difficult to predict [12]. Indeed, in this case, while the diagnosis of sugammadex anaphylaxis was made without significant delay, it was not immediately suspected for these reasons.

Sugammadex anaphylaxis may occur without prior sugammadex exposure, which can lead to delays in diagnosis [12,16]. In two prior case studies, five out of six cases of anaphylaxis had no previous exposure to sugammadex [17,18]. Sugammadex is a modified gamma cyclodextrin, and cyclodextrins are used as food preservatives, drug carriers, and in commercial products, so exposure to or ingestion of these may lead to sensitization [12,16]. 

A systematic review by Arslan et al. [18] demonstrated that hypotension and hypoxia were present in 93.9% and 45.4% of cases of sugammadex anaphylaxis, respectively [19]. A systematic review by Zecic et al. [19] demonstrated symptoms of hypotension (92%), erythema (76%), desaturation (40%), swelling/edema (28%), and wheezing (28%) as the most common [20]. Finally, a retrospective review of general perioperative anaphylaxis demonstrated signs of hypotension (46%), bronchospasm (18%), tachycardia (9.8%), oxygen desaturation (4.7%), and bradycardia (3%) [14].

The diagnosis of sugammadex anaphylaxis was made here based on the acute presentation within minutes following sugammadex administration with simultaneous involvement of the skin (e.g., urticaria) and mucosal tissue (e.g., angioedema) following initial respiratory compromise and hypotension, in accordance with the World Allergy Organization guidelines [20]. Notably, no other drugs had been administered within a reasonable timeframe to have served as the anaphylaxis-inciting agent. Additionally, a tryptase level was drawn at 75 minutes and was significantly elevated at 83.8 ng/ml. This degree of elevation, combined with a normalized tryptase level at 15 hours, strongly supports the diagnosis of anaphylaxis. Finally, this patient had evidence of anaphylactic shock, with an elevated lactate of 4.01 at 23 minutes after sugammadex administration.

## Conclusions

Intraoperative sugammadex anaphylaxis is a very rare but life-threatening event. While the acute, severe instability posed challenges here, the quick resolution and favorable recovery of this patient demonstrate the importance of swiftly diagnosing and treating intraoperative sugammadex anaphylaxis. The diagnosis, however, poses quite a challenge to the clinician, especially since it tends to occur at the end of an anesthetic, which is uncommon for intraoperative anaphylaxis; it happens during a time of expected rapidly fluctuating hemodynamics, and can occur without prior sugammadex exposure. This triad further increases the potential for patient morbidity and mortality and highlights the need for anesthesiologists to remain vigilant for signs of anaphylaxis after administration of sugammadex, possibly even more so in sugammadex-naïve patients, and especially since sugammadex administration will be more frequent as it comes off patent. This report may serve as a reminder to consider sugammadex anaphylaxis when undifferentiated shock is encountered in the emergence or postoperative phases of care.
